# New Jersey State Dental Society

**Published:** 1891-07

**Authors:** Charles A. Meeker

**Affiliations:** Secretary


### NEW JERSEY STATE DENTAL SOCIETY.—TWENTIETH ANNUAL SESSION.

(Continued from page 406.)

*Thursday, July 17, 1890.—Evening Session.*

The Secretary called the roll.

The election of officers was then proceeded with.

The following officers were elected:

George Emery Adams, D.D.S., President; B. F. Luckey, D.D.S. Vice-President; Charles A. Meeker, D.D.S., Secretary; George C. Brown, D.D.S., Treasurer.

*Executive Committee.*—B. F. Luckey, D.D.S., Dr. Oscar Adelberg R. M. Sanger, D.D.S., C. W. F. Holbrook, D.D.S., S. C. G. Watkins, D.D.S.

*State Board of Examination and Registration in Dentistry.*—Frederick A. Levy, D.D.S., G. Carleton Brown, D.D.S., F. C. Barlow, D.D.S., E. M. Beesley, D.D.S., A. R. Eaton, D.D.S.

*Committee on Membership.*—J. W. Curtis, D.D.S., Dr. J. A. Osmun, Dr. J. L. Crater, I. M. Vandewater, D.D.S., W. E. Truex, D.D.S.

Dr. John S. Marshall, of Chicago, Ill., was then called upon to read his paper on "Aneurismal Tumor of the Right Alveolar Process," etc.

(For Dr. Marshall's paper, see page 367.)

*Dr. Palmer.*—I want to express, as a member of the New Jersey State Association, my personal gratification and satisfaction at having had the pleasure of listening to Dr. Marshall's paper. I am not in a position to discuss such a paper; indeed, it is hardly one that admits of discussion, but as a member of this Society I desire to express my personal thanks to Dr. Marshall, and I believe I express the sentiment of a number of others.

*Dr. Peirce.*—The paper of Dr. Marshall very clearly states the conditions and the results, but does not admit of very much discussion. I have had great pleasure in listening to it, as we all must have had.

On motion, the paper was passed.

Dr. Charles B. Atkinson then read his paper on "Prophylaxis in the Field of the Dental Surgeon."

(For Dr. Atkinson's paper, see page 371.)

Adjourned until Friday, July 18, 1891, at 10 o'clock A.M.

*Friday, July 18, 1891.—Morning Session.*

Dr. B. F. Luckey presented the following report from the Clinic Committee:

Dr. L. M. Warner representing Dr. George Evans, of New York, inserted two of Dr. Evans's seamless gold crowns; one, an ordinary superior bicuspid, the other, an adjoining molar in which decay had penetrated chiefly below the gum line; the loss of tissue at that point was restored by building up with amalgam so as to make a solid foundation for the attachment of the crown. Dr. Seymour inserted a gold filling for the benefit of the Board of Examiners. Dr. Sanger, of Orange, very beautifully and very quickly inserted a Logan crown, and Dr. Z. T. Sailer, of New York, exhibited a split pin crown and some instruments of his own designing for the fitting and adaptation of the collars to the necks of the teeth. Dr. W. P. Richards, of Orange, also inserted a filling. Dr. Faught, of Philadelphia, who was to exhibit his electrical instrument for the insertion of gutta-percha into the roots of teeth, was unable to do so because a portion of the apparatus was damaged, and Dr. Adelberg, of Elizabeth, inserted an amalgam filling.

Dr. Adams, of the Exhibit Committee, reported as follows:

Your committee has done its best to have the exhibits a credit to the Society, and while not as successful as was the same committee last year, yet they have done all they could. There have been eleven exhibitors, and there were several other applications for information concerning space, all of which were responded to, and there were some who expressed an intention of exhibiting who did not do so.

*Dr. C. W. F. Holbrook.*—The Committee on Crown- and Bridge-work have nothing of importance to report, but they heartily endorse the crown- and bridge-work, more particularly in small cases. Dr. Bonwill presented some very interesting exhibits; they were a little out of the line of bridge-work proper, but they served very nicely to take the place of bridge-work. They were small gold plates and are highly recommended by the committee.

The Committee on Materia Medica, through Dr. Luckey, chairman, stated that they had no report.

The President then called up the further discussion of Dr. Atkinson's paper.

DISCUSSION OF DR. ATKINSON'S PAPER ON "PROPHYLAXIS IN THE FIELD OF THE DENTAL SURGEON."

*Dr. W. S. Elliott.*—Following the suggestions of the essayist in such order as may be possible, we observe, first, the education of the mother in relation to her own health and that of her offspring. Here we admit the supreme importance of the subject, and it is here that the most advanced claims come to be asserted in the possibilities of prevention rather than of cure.

To lay the foundation of perfect tooth-structure, the mother should take cognizance of the faults of her own nature and be able through a proper *régime* to redeem those faults in the more perfect organization of her children. The field of observation is a broad one, and were it covered by true knowledge of requirements little need would there be for professional interference; but the circumstances of modern life—the inevitable struggle for existence—preclude the possibility of attaining to any but a moderate degree of acquisition, therefore authoritative advice and direction may not be undesirable or unheeded. The danger of over-specification is, however, to be guarded against, since rules of limitation in diet and environment may not infrequently fall short of that consummation which natural desire would more perfectly attain to. Living by rule tends to strongly concentrate the thoughts upon one's self, which makes life an artificial one and warps the sentiments and affections of one's nature. While moderate discrimination in the selection of food is proper, we would lay much stress upon mental influences of the mother in shaping the destiny of the new life. Impressible as is the fœtus *in utero*, care should be taken that the current of vital energy be not diminished or deflected to result in deformity or incompleteness. The higher qualities and affections of the mind, then, we would claim, are powerful agents to the production of a status of physical being to which we would aspire as our most exalted ideal. The body is largely a reflection of the mind, therefore we would hold that right-mindedness gives right bodiedness. To be whole is to be holy. Live the higher life. Drink to the soul of truth. "Be clean" is the common injunction applicable to all, whether in reference to habits of mind or to habits of body, and we can favor no higher law than this as of universal application.

Aside from these general indications and such as fall more especially to the province of the practical dentist is the immediate care of the teeth. The injunction here is of paramount importance, and if religiously observed little more need be done towards a complete prophylaxis. Special instruction in this direction is legitimate as facilitating the effort, and in correspondence with the later understanding of dental pathology we would modify our formulæ for tooth-powders, washes, etc. The brush is indispensable, but allow me to pass sentence against that most disgusting substitute, the felt brush; reeking with the filth of putrefaction, it is certainly unfit for the purpose recommended. An acceptable powder is composed of precipitated chalk, soap, tannin, salicylic acid, and sugar, with flavor to suit the taste. For an antiseptic wash we would recommend a solution of Carl Sieler's tablets. This has an alkaline reaction, and, being antiseptic, is highly preservative of a normal condition.

Preventive measures are not the less called for under conditions of infraction; but now restoration takes the first place, and after the attainment of possibilities in this direction the same foregoing rules of prophylaxis are to be observed for the maintenance of the restored conditions. But restoration implies not only the repair of the teeth through direct operative and medical effort, but the intelligent meeting of contingencies which interfere with systemic wholeness. The dentist should be a physician in all that the title implies, that he may at least take cognizance and make due estimate of outlying needs to further his more immediate aims.

In this respect the points referred to by the essayist may be deemed too remote and too numerous to be entered upon by the practical dentist, and as not being "within the field of the dental surgeon." Should, however, a more extended knowledge of dietetics be desired, we would refer to statistics of scientific demonstrations, emanating from our public institutions, showing the relations existing between food and physical development, the details of which would outreach the limits of this debate.

In connection with this, as important for consideration, is that of atmospheric influence. The well-recognized fact that impure air is the causative agent of many maladies, especially as occurring among children, would lead us to impart instruction to our patients as regards ventilation and the practice of disinfection. All this costs no more than the desire to do our highest duty, and to augment the welfare of those who would confide in our knowledge and judgment. This applies also to clothing, exercise, sleep, occupation, and general habits of life. Scientific direction in each implies a reasonable understanding covered by the curriculum of studies easily within the reach of all who would claim distinction as dental scientists.

Should the executant feel indisposed to encroach upon the field of the general practitioner in the treatment and care of systemic ailments, he cannot with consistency ignore the topical and general treatment of lesions of the mouth that fall under the head of oral surgery; and when he understands and realizes what are the sympathies and mutual relationship that exist between the teeth and other organs of the body, then will he the more fully appreciate the importance of a general as well as a topical prophylaxis. While the tendency is ever to specialize, yet a moderate breadth of attainment will enable him, at least, to admonish where admonition will be heeded; urge moderation where excess is apparent, and abstinence where judgment approves.

Thorough mastication is a most important factor in prophylaxis. It has a double significance in that it promotes perfect digestion and assimilation and imparts functional energy to the teeth, gums, and salivary glands. The evils arising from inattention to this physiological necessity is ever apparent in the depreciated standard of health of a large proportion of adults. We believe that this habit of neglect is largely due to the practice of continuing the administration of soft and pulpy food to children beyond the period when the young teeth should have vigorous exercise. It is scarcely to be wondered at that teeth are lost and health diminished under such adverse conditions.

The insertion of artificial substitutes in cases of loss of the natural organs is in strict compliance with the demands of prophylaxis, since not only is mastication more completely accomplished, but the functioning of the retained teeth is qualified to the standard of natural requirement. But here, as in the operation of filling or the application of therapeutic remedies, the highest skill and judgment is demanded that destructive elements may not enter where preservation is desired.

We cordially endorse the injunction laid upon the habit of the repeated use of instruments without thorough cleansing. We recognize no greater sin of practice than this, and be assured it is not a mere sentiment, for facts of observation have shown repeatedly that diseased conditions have arisen through contact of soiled instruments, especially noticed in cases of extraction and with a class of patients who are more commonly patrons of this department. It is my custom to dip the instruments into carbolized water prior to and after using, wiping them dry with chamois. Hot water, doubtless, is quite as efficient; but whatsoever method is adopted, keep ever this high injunction before the mind,—be clean.

There being no further speakers on the subject, on motion, the paper was passed.

The installation of officers was then proceeded with.

On motion, the following vote of thanks was passed:

*Resolved*, That the thanks of this Society be extended to Mr. James A. Bradley for his interest in this Society, and for placing tables, etc., at the disposal of the Executive Committee.

On motion, the following were also passed:

To the essayists who have attended the present meeting and read papers.

To the proprietor of the Coleman House and the exhibitors.

To the press for their endeavors to report the proceedings so faithfully (this resolution was responded to by Mr. Ross, of the Asbury Park *Journal*).

To the Legislative Committee for their efforts and kindness in securing the passage of the present law.

Adjourned *sine die*.

Charles A. Meeker, D.D.S.,
*Secretary*.

